# Pain Evaluation and Treatment in Children: A Practical Approach

**DOI:** 10.3390/children10071212

**Published:** 2023-07-13

**Authors:** Lorenzo Sansone, Cristina Gentile, Eleonora Agata Grasso, Armando Di Ludovico, Saverio La Bella, Francesco Chiarelli, Luciana Breda

**Affiliations:** Department of Pediatrics, University “G. D’Annunzio” of Chieti-Pescara, 66100 Chieti, Italy

**Keywords:** children, pain, pain management, assessment scales, analgesic agents, non-pharmacological techniques

## Abstract

Pain is the most common complaint reported by children who access the emergency departments, but despite its frequency and the availability of many international guidelines, it often remains underreported and undertreated. Recently, the American Academy of Pediatrics and the American Pain Society have reiterated the importance of a multidisciplinary approach in order to eliminate pain in children. In all pediatric settings, an adequate assessment is the initial stage in a proper clinical approach to pain, especially in the emergency departments; therefore, an increasing number of age-related tools have been validated. A wide range of analgesic agents are currently available for pain management, and they should be tailored according to the patient’s age, the drug’s pharmacokinetics and the intensity of pain. In order to facilitate the choice of the appropriate drug, a treatment algorithm based on a ladder approach can be used. Moreover, non-pharmacological techniques should be considered to alleviate anxiety and distress in pediatric age. This review aims to offer a simple but intuitive description of the best strategies for pain relief in children, starting with the prompt recognition and quantification of pain through adequate assessment scales, and following with the identification of the most appropriate therapeutic choice among the ones available for pediatric age.

## 1. Introduction

Pain is the most prevalent symptom in an emergency setting and continues to be one of the most difficult challenges for emergency care providers, particularly in children [1]. Pain accounts for up to 80% of pediatrics’ emergency department visits, with musculoskeletal injury as the most common complaint, followed by headache, abdominal discomfort, otalgia and sore throat [1].

Inadequate treatment of acute pain may have both short- and long-term repercussions. Indeed, neuroimaging studies have discovered long-lasting modifications in brain structure and connectivity correlating with the amount of acute pain exposure during the perinatal period and with subsequent cognitive and behavioral effects in adult age [1,2]. For this reason, in 2001, the American Academy of Pediatrics and the American Pain Society reiterated the need to eliminate pain in pediatric patients, using a multidisciplinary method [3].

Pain is a complex experience, resulting from the interaction between neural pathways and neurochemical mediators [2]. According to the “Association for the Study of Pain”, first provided in 1978 and recently revised, pain is defined as “a distressing sensory and emotional experience with actual or potential tissue injury” [4]. This definition highlights some new aspects of pain: (a) pain is always a personal symptom; (b) pain and nociception are not the same phenomenon; (c) pain may have side effects on social function and psychological aspects; and (d) verbal description is merely one of several ways to express suffering, particularly in young children.

Nociception refers to the process in which specialized fibers in the peripheral nervous system transmit impulses from the periphery to the brain’s higher centers through the spinothalamic tracts due to the connection of synapses within the spinal cord’s dorsal [5]. In the past, several studies have revealed the existence of distinct classes of nociceptors that, when activated by different stimuli, transmit signals through two basic types of afferent fibers: C fibers and A-delta fibers [6,7]. Individual responses to equal nociceptive stimuli vary widely in clinical observations. This phenomenon can be explained by physiologic and psychological variations between individuals, as well as by the varying interactions between the different neural systems [8]. Table 1 illustrates the physiological and clinical features of pain and its different transmission mechanisms. 

Despite the availability of many international guidelines, in clinical practice pain often remains underrecognized and undertreated [4]. In this review, we summarize the best strategies for pain relief in children including recognition, assessment, pharmacological and non-pharmacological treatment.

## 2. Pain Assessment

Evaluation of pain is the initial step for an appropriate clinical approach to pediatric pain management; hence, validated age-related pain assessment tools have been developed [9]. These include observational–behavioral measures, which reflect the patient’s reaction to pain, and self-reported scales based on the patient’s quantification of pain; these are the most reliable methods. 

**Figure 1:**
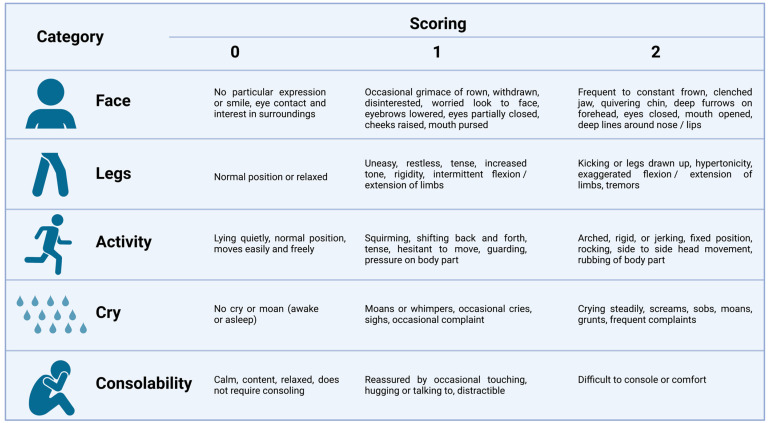
The Face, Legs, Activity, Cry and Consolability (FLAAC) scale for pediatric pain.

Observational–behavioral scales have been developed for younger children (<7 years) or cognitively impaired patients. The most commonly used, especially in the evaluation of postoperative pain, are the Children’s Hospital of Eastern Ontario Pain Scale (CHEOPS), Face, Legs, Activity, Cry and Consolability (FLAAC) and the Children and Infants’ Postoperative Pain Scale (CHIPPS) (Figure 1) [10]. 

The “COMFORT” scale, initially used in a pediatric critical care setting because of its complexity, is currently used also in the postoperative period [11].

**Figure 2:**
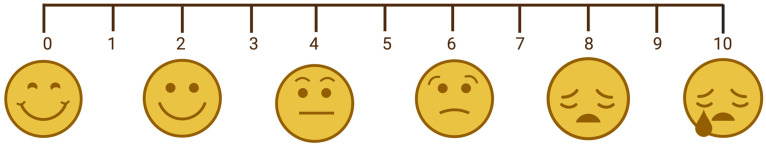
The Wong–Baker FACES scale for pediatric pain.

Children between three and seven years of age can articulate the intensity of pain; at this age, both observational and self-evaluation scales can be utilized. One of the most important is the Wong–Baker FACES^®^ Pain Rating Scale, represented by a series of faces from 0 (smiling face) to 10 (crying face), with 10 referring to the most intense pain. After a brief explanation, the child may choose the most representative face (Figure 2).

In 1996, a modified version of the Objective Pain Scale (OPS) was proposed by Wilson and Doyle, in which posture assessment replaced blood pressure (Modified Objective Pain Scale—MOPS), allowing an evaluation also by the patient’s parents [12].

In clinical practice, the most commonly used pain assessment tool is a numerical scale; however, this method needs an appropriate communication ability, which is only expected in school-age children (>8 years), since at that age they can generally comply with numerical rating or visual analogue scales [13]. At this age, the most used scales are the “Numeric Rating Scale” (NRS), the “Faces Pain Scale—Revised” (FPS-R) and the “Visual Analogue Scale” (VAS).

The VAS and NRS consist of assigning a value from 0 to 10 on horizontal lines, where 0 means no pain and 10 is used to refer the maximum pain [10]. Table 2 summarized the most often used pain scales in pediatric settings.

With the improvement of technology, electronic scales are becoming available for pediatric pain assessment, and their use may increase patients’ cooperation and lead to better pain management [14]. Furthermore, new diagnostic methods for an objective assessment of pain such as the measurement of skin conductance changes, “Analgesia Nociception Index” (ANI) and “Newborn Infant Parasympathetic Evaluation Index” (NIPE), have been developed in recent years; however, additional studies are needed [10].

## 3. Pharmacological Treatment of Acute Pain

**Figure 3:**
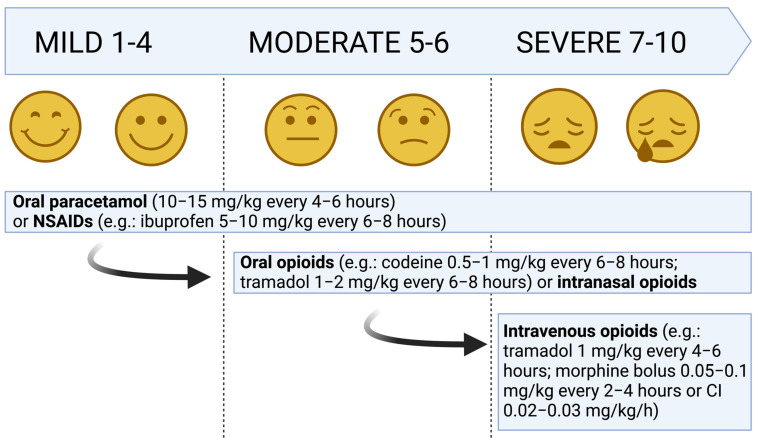
Step-up approach for the treatment of pediatric pain.

Currently, a wide range of analgesic agents are available for children and the choice of the drug depends on the child’s age, the drug’s pharmacokinetics and the intensity of the pain. A protocol instituted in the Emergency Department should indicate the administration of the best analgesic treatment for the severity and type of pain and should be started at the triage [15]. The evidence-based guideline from the European Society for Emergency Medicine is one of the few guidelines published concerning pain management both in children and in adults in emergency departments [16]. An algorithm has been developed with a range of flexible alternative options in order to meet the needs of individual settings (Figure 3): (a) for mild pain, acetaminophen is the drug of choice, with the possibility to use non-steroidal anti-inflammatory drugs (NSAIDs); (b) for moderate pain, acetaminophen is still the best drug, alone or with NSAIDs and oral opioids; and (c) in severe pain, first-line treatment relies on opioids such as morphine or fentanyl [16].

### 3.1. Acetaminophen

In children, acetaminophen is frequently the initial treatment for mild to moderate pain. A central effect of acetaminophen is the activation of descending serotonergic pathways. Its exact mode of action has not yet been determined, but it likely involves the inhibition of prostaglandin H2 synthesis; other possible pathways include the inhibition of cannabinoid-mediated activity or cyclooxygenase 3 (COX-3) [17]. Acetaminophen can be given intravenously, orally and rectally. The therapeutic plasma acetaminophen level for pain reduction is usually estimated as 10 mg/L [18]. Infants and neonates are known to have a reduced clearance of acetaminophen; thus, the maximum dosage should be reduced in children with less than 3 months of age (Table 3) [3,18,19].

Maximum serum concentrations occur four hours after rectal administration, whereas the intravenous route has a better dosing reliability, a fast start of action (within 5 min) due to less pharmacokinetic fluctuation and the ability to achieve peak cerebrospinal fluid levels in about an hour [3,18].

Infrequent adverse effects are associated with acetaminophen. However, hepatotoxicity in minors that took routine doses for 2–8 days has been reported. Although it is not possible to predict which subjects are at a higher risk of having liver damage, the maximum dose intake should be limited to three days [3].

### 3.2. NSAIDs

NSAIDs are prescribed for mild to severe pain in children because of their opioid-sparing action [3]. Their mechanism of action is the inhibition of the cyclooxygenase-2 (COX-2) that converts arachidonic acid to thromboxane and prostaglandins. The selective inhibition of COX-2 is associated with less gastrointestinal intolerance. Prostaglandins are stunning vasodilators and proinflammatory factors that stimulate the production of nociceptors [20]. Moreover, unlike acetaminophen, NSAIDs are also considered to have a peripheral anti-inflammatory role [3]. 

The main routes of NSAIDs administration and dosages are summarized in Table 4. NSAIDs have been associated with several adverse events including gastritis, gastrointestinal bleeding and kidney failure; however, these are uncommon in the pediatric age. Due to its association with Reye’s syndrome, the use of aspirin in children has decreased in recent years and is limited to specific rheumatologic disorders [3,20].

#### 3.2.1. Ibuprofen

Ibuprofen is the most used NSAID in clinical practice; indeed, it has been shown to be more efficient than acetaminophen and as good as the combination of acetaminophen and codeine for the management of acute pain associated with injuries to the musculoskeletal system, including certain limb fractures. Therefore, it can be used as a first approach in the treatment of migraines in children due to its faster pain relief compared to paracetamol [21].

For acute pain, the suggested dose of ibuprofen is 5–10 mg/kg every 6–8 h, with a maximum daily intake of 30 mg/kg. The peak of plasmatic concentration is reached 45–60 min after the administration to an empty stomach, whereas absorption is slower and variable if administered after a meal [21]. 

Ibuprofen is the least toxic NSAID for gastrointestinal adverse events in adults and children [22]. However, it should be administered carefully in pediatric patients with pre-existing kidney diseases and should not be administered to children with less than 3 months of age [3,21].

#### 3.2.2. Naproxen

Naproxen sodium is a common antipyretic and antirheumatic NSAID used in the pediatric population. This drug has a more prolonged elimination half-time than most NSAIDs, making it an effective analgesic for postoperative pain [23]. It has been reported that postoperative oral administration of naproxen reduces pain more effectively than oral acetaminophen (Table 4) [23].

#### 3.2.3. Diclofenac

Diclofenac is a safe and effective analgesic drug for perioperative pain in pediatric patients with a good safety profile; it is available in tablet, suppository and parental form [3]. The recommended dose is 1 mg/kg/day in patients between 1 and 12 years of age.

#### 3.2.4. Ketoprofen

Ketoprofen is a widely used NSAID in children for postoperative pain. It has been reported that there were better improvements in pediatric patients treated with ketoprofen versus placebo; no significant side events have been documented with the exception of increased intra-operative blood loss in ketoprofen-treated patients [24].

#### 3.2.5. Ketorolac

The first NSAID used to treat adult postoperative pain was ketorolac and, despite not being approved for use in the pediatric population, it has been studied in the pediatric population. Children require higher relative dosages of ketorolac than adults due to its higher reported volume of distribution [3,24]. The most important adverse effects are bleeding and nephrotoxicity. The risk of upper gastrointestinal bleeding seems to be higher compared to ibuprofen or other NSAIDs, such as indomethacin and diclofenac. Ketorolac should be avoided in patients with risk factors for renal dysfunction, such as dehydration and hypo-perfusion [25]. Several studies that examined the use of ketorolac in children in the perioperative setting showed that it was better tolerated than morphine, with a similar efficacy [24]. Therefore, the short-term use of ketorolac in the Emergency Department, alone or in association with paracetamol and opioids, is considered useful and safe, and its sublingual administration may be a valid alternative for moderate to severe pain management in the absence of vascular access [24].

### 3.3. Opioids

Opioids are typically prescribed to children with intermediate to severe acute nociceptive pain or acute pain that is resistant to treatment with non-opioid drugs. The natural alkaloids are also known as opiates, and include morphine and codeine, while synthetic derivatives include heroin, fentanyl, hydromorphone, methadone, buprenorphine and others [26]. Opioids act by binding specific cell receptors (e.g., µ, δ and κ) found predominantly in the brain, though these are also located in blood vessels, the heart, lungs, gut and mononuclear cells [26]. Opioids have a variety of clinical effects other than pain relief, including euphoria, changes in mood, drowsiness and mental clouding. A peculiar characteristic of the pain-relief effect induced by these molecules is the conservation of the state of consciousness. The route of opioids administration is dependent on pain severity and the condition of the patient (Table 5) [16]. In recent years, opioid transdermal delivery systems (TDDSs) have been developed with some advantages for opioid administration in children. TDDSs avoid blood peaks, allowing steady and continuous drug delivery and a reduction in side effects, while also improving patient compliance [27,28].

The most common adverse events include respiratory depression, decreased blood pressure, sedation, nausea, vomiting and decreased gastrointestinal motility with constipation [3,26,28]. Severe respiratory depression is unusual when intravenous opioids are titrated carefully with standard doses. To reverse unwanted opioid effects, opioid receptor antagonists, such as naloxone and naltrexone, are available [1,3,26].

#### 3.3.1. Morphine

Morphine is the prototype opioid that was first isolated from opium in 1804 [29]. Intravenous morphine is often the first drug used in most Emergency Departments to treat severe pain [29]. In addition, it is frequently administered via infusion or patient-controlled delivery following major surgery. Indeed, oral morphine has a relatively low bioavailability compared to IV routes, a slower effect and a complex pharmacokinetics [29]. A study focusing on morphine use in the postoperative period in pediatric patients did not clarify if there are differences in its adverse effects profile compared to other intravenous opioid drugs [30]. In a prospective study, oral morphine had reduced pain by 80% in children with limb fractures [31]. Also, a randomized controlled trial compared oral morphine versus oral ibuprofen in children with uncomplicated limb fractures, but did not find any differences in pain relief; however, the group treated with oral morphine experienced significantly more adverse effects [32].

#### 3.3.2. Codeine

Codeine is a natural derivative of opium and is considered to have a potency of one-tenth compared to morphine. It is converted to morphine by the cytochrome P450 CYP2D6, and, according to the amount of morphine produced, patients are divided into poor metabolizers if almost no codeine is converted and ultrarapid metabolizers, resulting in unpredictably high plasma concentrations [16,33]. Due to this, the European Medicines Agency banned the use of codeine for patients under the age of 12 in 2013 and extended the prohibition to those undergoing tonsillectomy and/or adenoidectomy for obstructive sleep apnea up to the age of 18 [33]. It has also been proposed that protocols substitute oral morphine for codeine [34].

#### 3.3.3. Fentanyl

Fentanyl is a purely synthetic, short-acting opioid, utilized for acute pain management and considered to be 50–100 times more potent than morphine [3].

It can be administered by intravenous or intranasal routes, and, more recently, by transdermal patches.

Intranasal fentanyl has a greater safety profile than intravenous morphine, without any difference in the effectiveness of pain control [3]. Transdermal fentanyl has been studied in children with chronic pain secondary to malignant and non-malignant diseases and was proved to be a valid alternative to more invasive administrations [33].

A Cochrane review concluded that intranasal fentanyl is well tolerated and effective in the treatment of moderate/severe acute pain in children [35]. Moreover, fentanyl has minor side effects on the cardiovascular system when compared to morphine [36].

#### 3.3.4. Tramadol

Similar in structure to morphine and codeine, tramadol is a centrally-acting drug for moderate to severe pain. Its opioid activity results from low-affinity binding to μ-opioid receptors; furthermore, it inhibits serotonin uptake, with a direct serotonin-releasing action [33]. Tramadol has been associated with respiratory depression after tonsillectomy operations in children with ultrarapid CYP2D6 metabolizers. For this reason, the FDA contraindicated the use of tramadol in patients under 12 years old and in patients under 18 years old after tonsillectomy or adenectomy [28].

#### 3.3.5. Ketamine

Ketamine is an N-methyl-D-aspartate antagonist that prevents the central sensitization of nociceptors and inhibits peripheral nociception [3]. It may be used to provide anaesthesia, sedation or analgesia. The effects of ketamine are dose-dependent, with analgesia without sedation occurring at around 10–30% of the dissociative dose. 

In a meta-analysis of four randomized clinical trials, intranasal analgesic-dose ketamine was found to be as efficacious as intranasal fentanyl for the management of moderate to severe acute pain for children in the Emergency Department; the most frequent adverse events were dizziness, unpleasant taste and drowsiness [37].

### 3.4. Nitrous Oxide

Nitrous oxide (N2O) is a useful agent for pediatric procedural sedation, because it provides a rapid onset and offset of sedation [1]. A nasal or face mask or muzzle is used to self-administer N2O via a demand valve system or continuous flow [38]. It can be administered as 50% or 70% with oxygen for both analgesia and sedation; the higher dose provides a similar sedation depth with no increase in adverse events [39].

Despite its efficacy, some studies indicate that N2O alone provides limited pain relief during extremely excruciating procedures, such as fracture reduction [40]. The most frequent adverse events reported are nausea, vomiting, agitation and dizziness [3].

## 4. Non-Pharmacological Treatment of Pain

In children, needle procedures such as venipuncture or intravenous cannulation are related to anxiety and distress, which are often reported as the worst experience of the day for hospital admission. If not appropriately managed, they are an important factor of increased pain perception on future procedures. It has been reported that non-pharmacological measurements may reduce pain and anxiety caused by invasive procedures, such as venipuncture, in children and adolescents [1]. A non-pharmacological approach to pain includes psychological, behavioral and physical interventions used as adjuncts to pharmacological treatment and consist of physical comfort measures and distracting activities (Table 6) [1].

## 5. Conclusions

Pain is a frequent complaint in pediatric age patients, and its management remains one of the most important challenges for pediatricians, especially in emergency settings. 

Various considerations should be made to select the most appropriate analgesic medication. The initial assessment of pain represents the key stage in its management and relies on appropriate scales tailored to the patient’s age and cognitive level. After recognizing the severity of the pain, an appropriate treatment should be selected, considering all non-pharmacological interventions that may help reduce anxiety and distress. Due to their safe profile, both acetaminophen and NSAIDs are often used as the first-line treatment for mild to moderate pain. On the other hand, opioids remain the cornerstone of treatment for acute procedural, postoperative and chronic pain in the pediatric population, with the intravenous route being the first choice in an emergency setting. Intranasal fentanyl, which acts quickly within 10 min of administration, is indicated in the management of acute pain for procedural analgesia. However, additional research is necessary to enhance the safety profile of the principal analgesic drugs in the event of overdosing or intoxication in children to reduce the risks of hepatotoxicity, gastrointestinal hemorrhage and renal injury. 

Once the treatment has been administered, a periodic reassessment is mandatory to better define the diagnosis, to modify the current regimen according to the patients’ needs and to define the ultimate treatment.

## Figures and Tables

**Table 1:** Physiological and clinical features and different mechanisms in pain transmission.

	Nociceptive Pain		Neuropathic Pain
	Somatic Pain	Visceral Pain	
Origin	Superficial receptors	Visceral receptors	Nerves
Location and distribution	Superficial (skin and subcutaneous tissue) Well-localized Not referred	Deep (e.g., from muscle/bone/fascia/periosteum) Poorly localized Often referred	Nerve structures (e.g., trigeminal nerve) Radiating or specific
Transmission	A-delta-fibers	C-fibers	Dermatomal (periphery) or non-dermatomal (central)
Type of pain reported	Pinprick, sharp or stabbing	Pressure, sharp or ache	Tingling, prickling, lancinating or burning

**Table 2:** The most often used pain scales in pediatrics.

Acronym	Age Range
CHIPPS	Under 5 years
CHEOPS	1–7 years
FLACC	2 months–7 years
OPS and MOPS	8 months–13 years
Poker Chip Tool	From 3 years
Oucher Scale	3–12 years
Wong–Baker FACES^®^ Pain Rating Scale	From 3 years
FPS-R	From 4 years
VAS	From 5 years
NRS	From 8 years

Abbreviations: CHIPPS (Children and Infants Postoperative Pain Scale); CHEOPS (Children’s Hospital of Eastern Ontario Pain Scale); FLACC (Face, Legs, Activity, Cry and Consolability); OPS/MOPS (Objective Pain Scale/Modified Objective Pain Scale); FPS-R (Faces Pain Scale—Revised); VAS (Visual Analogue Scale); and NRS (Numeric Rating Scale).

**Table 3:** Routes and dosages for acetaminophen administration.

Route	Newborns	Children < 40 kg	Children ≥ 40 kg	Maximum Dose
Intravenous	20 to 40 mg/kg/day	7.5–15 mg/kg every 4–6 h	325–650 mg every 4–6 h. May give up to 1000 mg in one loading dose	75 mg/kg/day for infants 100 mg/kg/day for children < 40 kg. 3–4 g/day for ≥40 kg.
Oral	25 to 30 mg/kg/day in preterms of 30 weeks of gestational age; 45 mg/kg/day in preterms of 34 weeks of gestational age; 60 mg/kg/day in term neonates	10–15 mg/kg every 4–6 h		
Rectal		Loading dose of 30 mg/kg; then, 15–20 mg/kg every 6 h		

**Table 4:** Routes and dosages for NSAIDs administration.

Non-Steroidal Anti-Inflammatory Drugs (NSAIDs)	Route	Dose
Ibuprofen	Oral	5 to 10 mg/kg, every 6–8 h
Naproxene	Oral	5 to 7.5 mg/kg, every 12 h
Diclofenac	Oral Intravenous	0.3 to 1 mg/kg, every 8 h 0.3 to 1 mg/kg, every 12–24 h
Ketoprofen	Oral Intravenous	0.3 to 2 mg/kg, every 8–12 h 0.3 to 2 mg/kg, every 8–12 h
Ketorolac	Intravenous	0.5 mg/kg, every 6–8 h

**Table 5:** Routes and dosages for opioids administration.

Class	Drug	Route	Dose
Weak opioids	Codeine	Oral/Rectal	0.5–1 mg/kg, every 6–8 h
	Tramadol	Oral	1–2 mg/kg, every 6–8 h
		Intravenous	1 mg/kg, every 4–6 h
Strong opioids	Morphine sulphate	Oral	0.15–0.3 mg/kg, every 4 h
	Morphine hydrochloride	Intravenous	Bolus: 0.05–0.1 mg/kg, every 2–4 h CI: 0.02–0.03 mg/kg/h
	Fentanyl	Intravenous	Bolus: 0.001–0.002 mg/kg CI: 0.001 mg/kg/h
		Intranasal	0.0015–0.002 mg/kg/dose

Abbreviations. CI: Continuous infusion.

**Table 6:** Non-pharmacological treatment of pain.

Approach	Type	Indication
Psychological	Sharing information about procedures	Procedural pain Postoperative pain
Distractive techniques	Active distraction ● Interactive toys ● Virtual reality ● Controlled breathing ● Guided imagery and relaxation	Procedural pain Postoperative pain
	Passive distraction ● Auditory Distraction: Music ● Audiovisual Distraction: Television	Procedural pain Postoperative pain
Non-distractive techniques	Transcutaneous Electrical Nerve Stimulation	Postoperative pain
	Cognitive Behavioral Therapy	Postoperative pain Procedural pain
	Acupressure	Minor trauma
	Cryotherapy and heat therapy	Musculoskeletal injuries
	Traction and bracing	Fractures Traumas

## Data Availability

Not applicable.
